# Supplementary material for: Impact of global short-term landscape fire sourced PM2.5 exposure on child cause-specific morbidity: a study in multiple countries and territories
Source: Nat Commun. 2025 Oct 22;16:9347. doi: 10.1038/s41467-025-64411-0 (PMC12546595; doi:10.1038/s41467-025-64411-0)
Supplement: Supplementary file 1 — Supplementary information [file 41467_2025_64411_MOESM1_ESM.pdf]

Method S1 Hospital admission data collection and organization

The individual hospital admission data were obtained from the New South Wales (NSW, covering 32% of the population in Australia) Admitted Patient Data Collection (APDC) in NSW of Australia (covering 100% of hospitalization records) [1], the Hospital Information System of the Unified Health System (SIH-SUS) in Brazil (covering 70–80% of hospitalization records) [2], the Hospital Morbidity Database (HMDB) in Canada (covering all hospitalization records) [3], the Ministry of Health (MOH) in Chile (covering about 70% of hospitalization records) [4], the National Minimum Dataset in New Zealand (covering all hospitalization records) [5], the National Health Insurance Research Database (NHIRD) in Taiwan (covering all hospitalization records) [6], the Ministry of Public Health (MoPH) in Thailand[7] (covering all hospitalization records). These individual hospital admission data were aggregated by age (0-4, 5-9, and 10-19 years), sex (female or male), disease cause defined by the International Classification of Diseases 10th [ICD-10] codes (all-causes, infectious diseases [A00-99 or B00-99], cancer [C00-97], diabetes [E10-14], neurological system disorders [G00-99], cardiovascular diseases [I00-99 or G45-46], respiratory diseases [J00-99], digestive diseases [K00-93], and renal diseases [N00-19]). These outcomes were selected to comprehensively assess the diverse potential health impacts of LFS $PM_{2.5}$, as ambient air pollution has been shown to affect these health outcomes in children [8]. The records from Taiwan were not available for cardiovascular, neurological, diabetes, cancer, or renal diseases due to the limited numbers. Consequently, an integrated multi-country dataset of daily age-, sex- and cause-specific hospitalization counts for cause-specific disease in 1012 communities across seven countries or territories was yielded for subsequent analysis.

Method S2 Community-specific model

Community-specific LFS-hospital admission associations were evaluated using a quasi-Poisson regression distributed lag non-linear model as follows: as follows:

$$Y_{it} \sim poisson\ (\mu_{it})\ \log(\mu_{it}) = cb(LFS\ PM_{2.5}, lag = 14) + cb(Temperature_{it}, lag = 21) + ns(Relative\ humidity_{it}, df = 3) + ns(Time_{it}, df = 7/year) + DOW_{it}$$

Where Y_{it} is the number of all-cause and cause specific hospitalisations in community *i* on day *t*; *cb(LFS* $PM_{2.5}$*,lag=14)* is the crossbasis function for LFS $PM_{2.5}$ over 0-14 days [9], where the exposure response relationship was modelled with linear function and lag response relationship was modelled using natural cubic spline with two internal knots placed equally in the log scale of lag days; *cb(Temperature,lag=21)* is the crossbasis function for temperature over 0-21 days, where the exposure response relationship was modelled using natural cubic spline with three internal knots at the 25th, 50th, and 75th centiles of the community-specific temperature distribution and lag response relationship was modelled using natural cubic spline with two internal knots placed equally in the log scale of lag days [10]; ns(Relative humidity, df=3) is a natural cubic spline for the moving average of relative humidity during 0-7 days with 3 degrees of freedom. To control for seasonal trend and long-term trend, a natural cubic spline with seven degrees of freedom per year and an indicator for the day of the week (DOW) were included in the model. We adjusted for temperature because it's a known confounder in the LFS $PM_{2.5}$-hospital admission associations. Previous studies have consistently included temperature in the models to reduce potential residual confounding effects.

Method S3 Modification effect of sex, age, and socioeconomic status

To assess effect modification by sex and age, we modelled sex- and age-specific hospitalizations separately within each community. Pooled community-level estimates were then compared across subgroups using a Wald-type test [11] to test for differences by sex and age. For socioeconomic status, community-level estimates were pooled within different groups of communities classified by socioeconomic status and also compared using a Wald-type test. These Wald-type tests took female, 5-9 years, high GDP level, and high-income country as the reference categories.

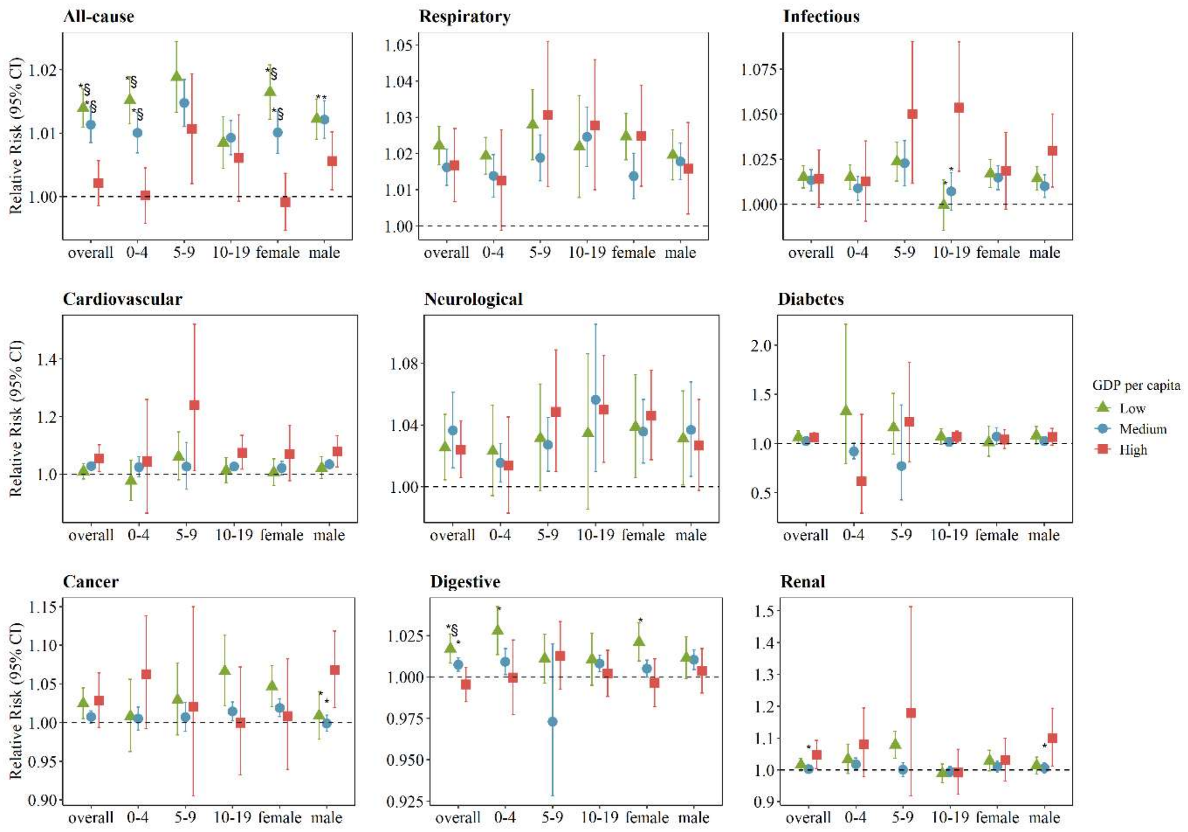

Figure S1. The cumulative relative risks of all-cause and cause-specific hospital admissions associated with 10 μg/m^3 increase in LFS $PM_{2.5}$ during lag 0-1 day, by levels of GDP per capita.

Data are presented as estimated relative risks (RRs) with 95% confidence intervals (CIs). Central points represent the estimated RRs, and error bars indicate the corresponding 95% CIs. Estimates are derived from a two-stage time-series analysis.

**P_unadjusted* < 0.05, P values are not adjusted for multiple comparisons. §*P_adjusted* < 0.05, P values are adjusted for multiple comparisons using the FDR method. The adjustment was implemented using the Benjamini-Hochberg (BH) method via the p.adjust function (method = "fdr") in R. All statistical tests were two-sided.

Abbreviations: CI, confidence interval; GDP, gross domestic product;

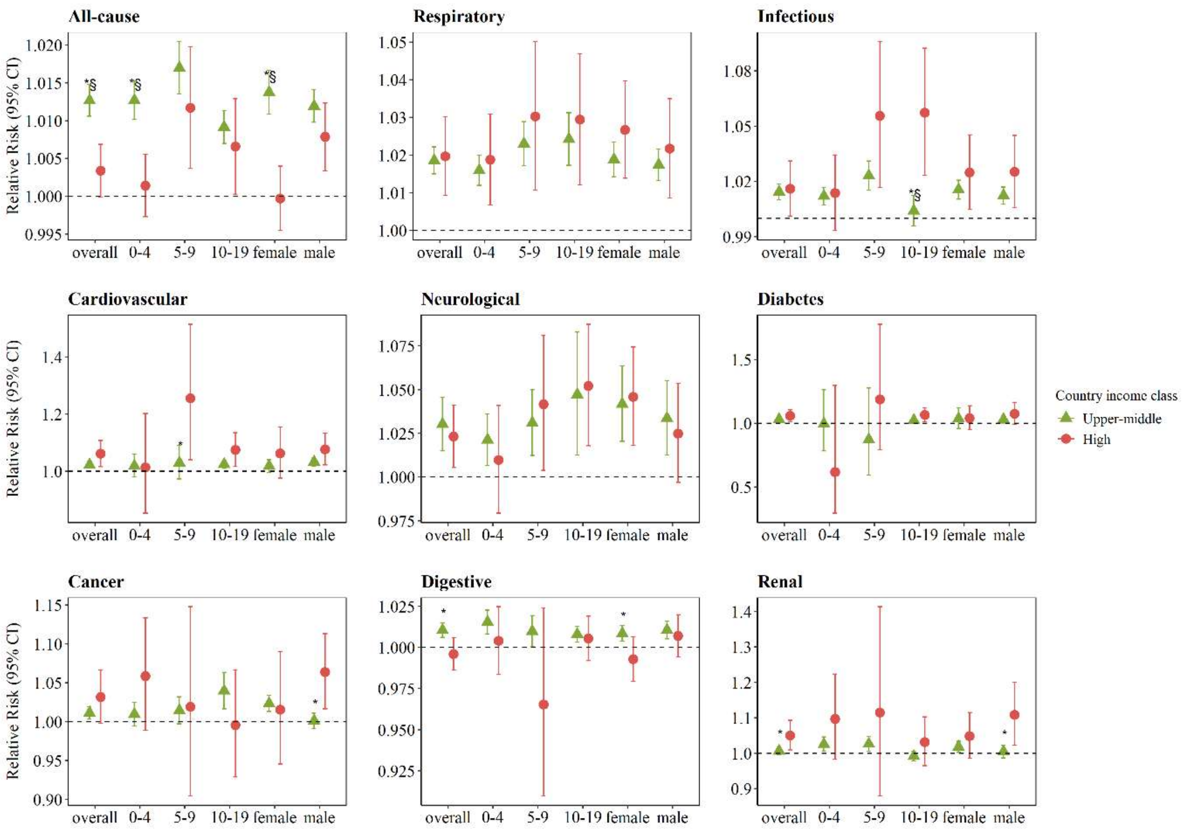

Figure S2. The cumulative relative risks of all-cause and cause-specific hospital admissions associated with 10 μg/m^3 increase in LFS $PM_{2.5}$ during lag 0-1 day, by country income class.

Data are presented as estimated relative risks (RRs) with 95% confidence intervals (CIs). Central points represent the estimated RRs, and error bars indicate the corresponding 95% CIs. Estimates are derived from a two-stage time-series analysis.

**P_unadjusted* < 0.05, P values are not adjusted for multiple comparisons. §*P_adjusted* < 0.05, P values are adjusted for multiple comparisons using the FDR method. The adjustment was implemented using the Benjamini-Hochberg (BH) method via the p.adjust function (method = "fdr") in R. All statistical tests were two-sided.

Abbreviations: CI, confidence interval

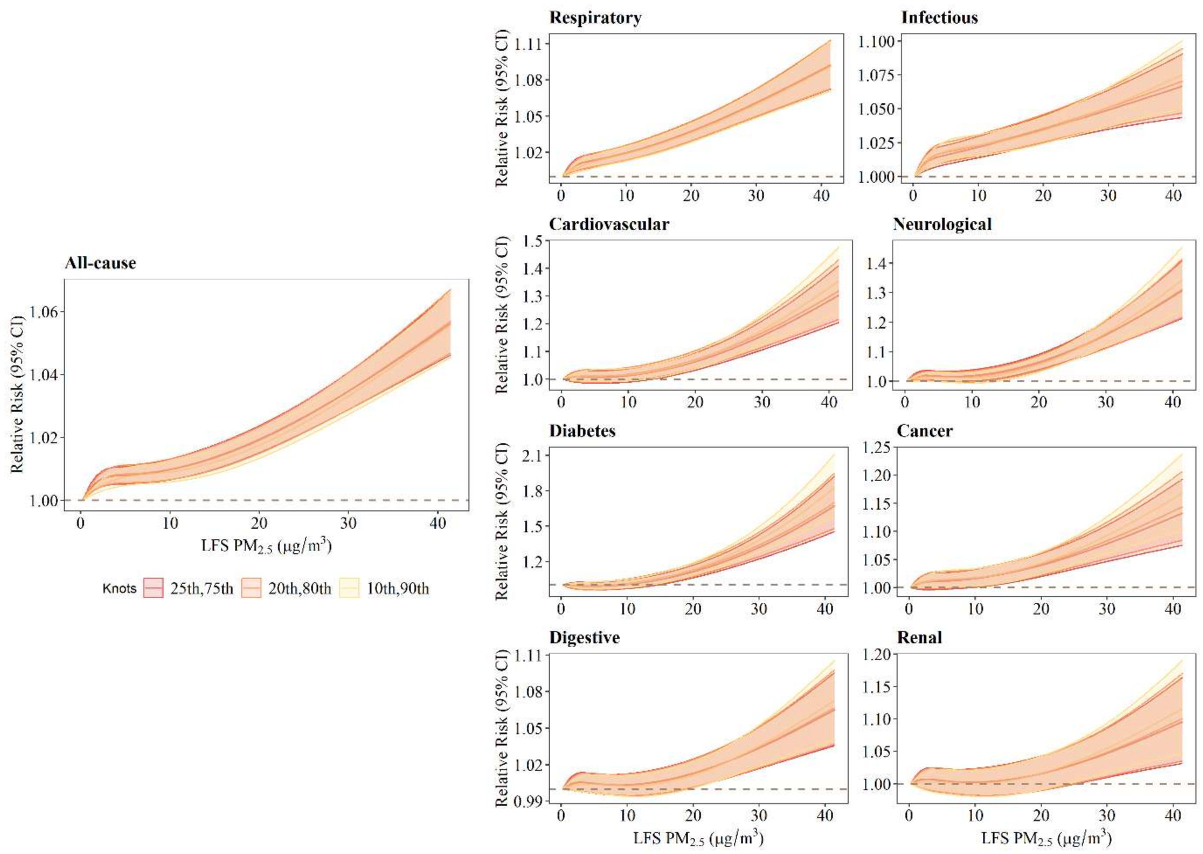

Figure S3. The cumulative exposure-response curves for the association between LFS $PM_{2.5}$ during lag 0-1 days and all-cause and cause-specific hospital admissions, estimated by models with different knots place for LFS $PM_{2.5}$.

Data are presented as estimated relative risks (central solid lines) with 95% confidence intervals (shaded areas). Curves were estimated using a two-stage time-series approach with a natural cubic spline. Three spline knot placements were used: at the 25th and 75th percentiles (dark red), 20th and 80th percentiles (orange), and 10th and 90th percentiles (light orange) of the LFS $PM_{2.5}$ distribution.

Abbreviations: CI, confidence interval

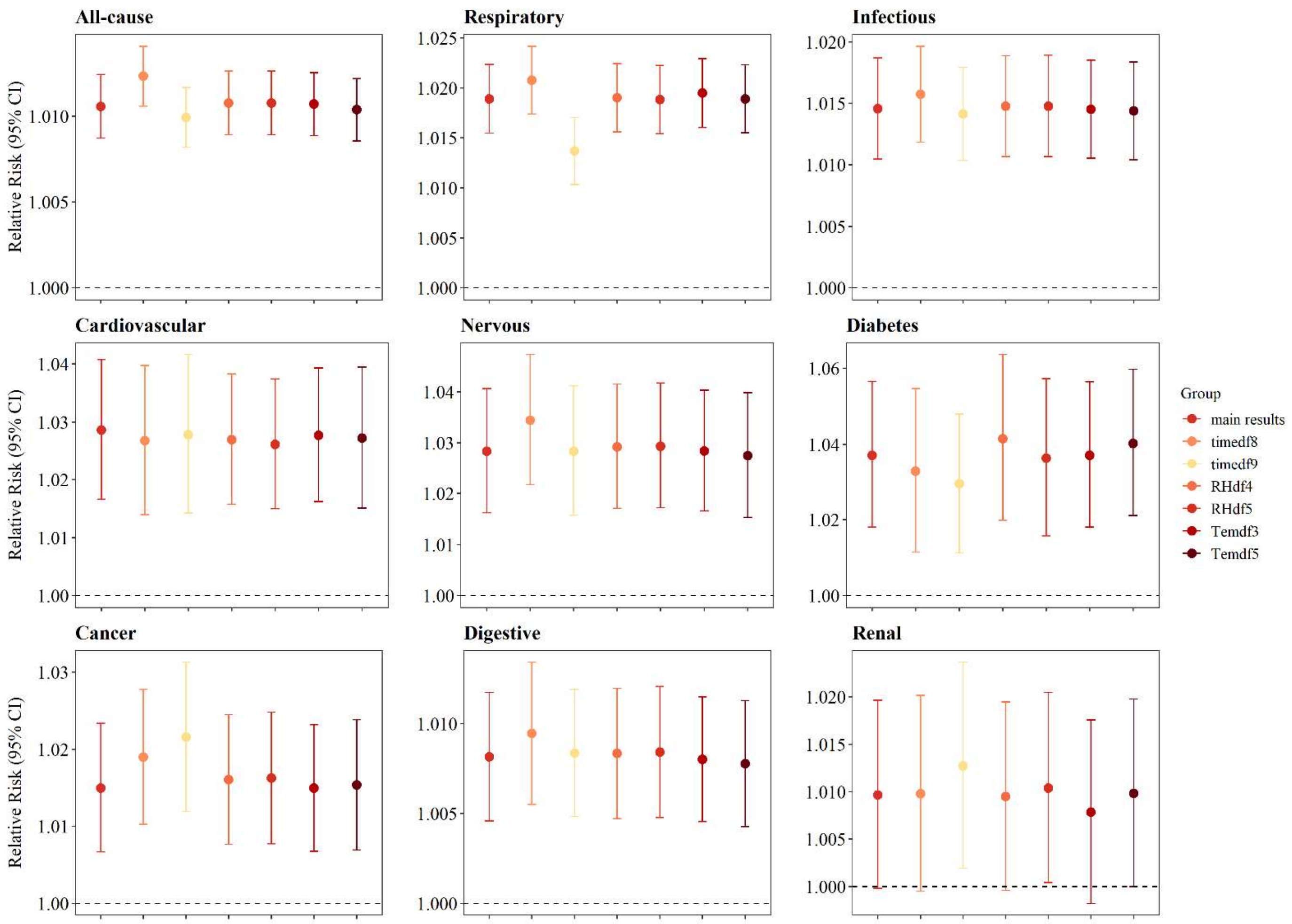

Figure S4. The cumulative relative risks of all-cause and cause-specific hospital admissions estimated by models with different degrees of freedom.

Data are presented as estimated relative risks (RRs) with 95% confidence intervals (CIs). Central points represent the estimated RRs, and error bars indicate the corresponding 95% CIs. Estimates are derived from a two-stage time-series analysis.

Abbreviations: CI, confidence interval

Table S1. Summary of the spatial unit, number of study locations, and estimated daily LFS $PM_{2.5}$ ($\mu g/m^3$) in seven countries/territories during the study period.

**Country /Territory**	**Spatial unit**	**No. of locations**	**Period**	**Area (km^2, median ± IQR)**	**Population (median ± IQR)**	**Hospitalization coverage**	**LFS $PM_{2.5}$ ($\mu g/m^3$)**	
							Median	IQR(25th,75th)
Overall	-	1012	2000–2019	5,384.1±11,870.9	126,707.5±224,614.7	-	1.2	3.0 (0.4, 3.4)
Australia (NSW)	SA3	83	2001–2019	391.0±6,760.3	68,201.6±57,171.8	100%	1.1	2.0 (0.5, 2.5)
Brazil	Immediate region	509	2007–2019	6,896.7±11,961.4	178,662.1±183,994.0	70-80%	2.2	4.9 (0.9, 5.7)
Canada	Second-level administrative divisions	256	2005–2019	4,177.6±16,152.1	44,831.6±69,945.2	100%	0.3	0.9 (0.1, 1.0)
Chile	Immediate region	15	2001–2019	38,092.9±47,932.4	667,100.4±715,203.8	100%	9.5	10.4 (5.1, 15.5)
New Zealand	Territory	66	2000–2019	2,791.9±4,435.0	30,283.3±36,075.8	100%	0.7	1.3 (0.3, 1.6)
Thailand	Province	77	2015–2019	5,604.1±6,052.3	622,255.1±539,806.0	100%	1.6	7.1 (0.6, 7.7)
Taiwan	Municipality	6	2000–2018	2,146.3±796.9	2,562,462.1±476,096.5	100%	1.1	1.3 (0.5, 1.9)

Abbreviations:SA3, Statistical Areas Level3. NSW, New South Wales

Table S2. The number of all-cause and cause-specific hospital admissions of children and adolescents aged 0-19 years across subgroups during the study period.

Subgroups	Hospital admission counts (n)								
	**All-cause**	**Respiratory**	**Infectious**	**Cardiovascular**	**Neurological**	**Diabetes**	**Cancer**	**Digestive**	**Renal**
**Overall**	67,947,787	13,814,221	6,593,189	503,667	1,110,667	209,674	858,784	4,843,877	642,461
**Country/Territory**									
Australia	5,405,114	661,689	203,708	31,245	139,157	21,750	48,081	479,231	24,396
Brazil	32,358,838	7,070,841	4,186,117	311,017	574,981	100,757	543,442	2,151,154	426,968
Canada	9,160,965	824,904	196,130	52,907	132,420	46,058	42,903	682,174	49,654
Chile	7,237,174	1,576,827	418,411	63,504	146,111	22,554	159,469	698,283	94,427
New Zealand	4,355,509	537,161	223,927	31,645	67,977	16,938	32,716	304,886	21,924
Thailand	4,799,084	1,724,415	1,062,059	13,349	50,021	1,617	32,173	281,972	25,092
Taiwan	4,631,103	1,418,384	302,837	-	-	-	-	246,177	-
**Sex**									
Female	31,443,165	6,775,232	3,293,244	242,480	558,996	100,523	443,282	2,455,039	299,887
Male	36,500,344	7,038,982	3,299,939	261,182	551,664	109,147	415,501	2,388,812	342,572
**Age**									
0-4	32,202,087	8,346,116	3,681,197	127,502	412,933	22,429	244,097	1,458,597	179,706
5-9	11,742,164	3,427,300	1,617,902	97,874	295,395	39,806	226,093	1,280,586	156,224
10-19	24,003,536	2,040,805	1,294,090	278,291	402,339	147,439	388,594	2,104,694	306,531
**Country income class**									
Upper-middle	23,552,691	3,442,138	926,602	115,797	339,554	84,746	123,700	1,712,468	95,974
High	44,395,096	10,372,083	5,666,587	387,870	771,113	124,928	735,084	3,131,409	546,487
**GDP**									
Low	21,126,159	5,302,439	2,962,321	149,500	269,403	44,274	247,487	1,279,550	219,684
Middle	29,496,793	6,668,490	3,052,001	248,065	523,414	88,272	495,090	2,271,969	335,991
High	17,324,835	1,843,292	578,867	106,102	317,850	77,128	116,207	1,292,358	86,786

Reference

1. Lam MK. How good is New South Wales admitted patient data collection in recording births? *Health Inf Manag* 2011; **40**(3): 12-9.
2. Nakamura-Pereira M, Mendes-Silva W, Dias MA, Reichenheim ME, Lobato G. [The Hospital Information System of the Brazilian Unified National Health System: a performance evaluation for auditing maternal near miss]. *Cad Saude Publica* 2013; **29**(7): 1333-45.
3. Amuah JE, Molodianovitsh K, Carbone S, et al. Development and validation of a hospital frailty risk measure using Canadian clinical administrative data. *CMAJ* 2023; **195**(12): E437-E48.
4. Department of Health Statistics and Information. 2023. https://deis.minsal.cl/#datosabiertos.
5. National Minimum Dataset (Hospital Events). 2022. https://www.health.govt.nz/publication/national-minimum-dataset-hospital-events-data-dictionary (accessed 11-20 2023).
6. Lin L-y, Warren-Gash C, Smeeth L, Chen P-C. Data resource profile: the National Health Insurance Research Database (NHIRD). *Epidemiology and Health* 2018; **40**.
7. Kliengchuay W, Srimanus W, Srimanus R, et al. The association of meteorological parameters and AirQ+ health risk assessment of PM(2.5) in Ratchaburi province, Thailand. *Sci Rep* 2022; **12**(1): 12971.
8. Liu K, Zhang H, Bo Y, et al. Ambient air pollution and Children's health: An umbrella review. *Atmospheric Pollution Research* 2024; **15**(6): 102108.
9. Gasparrini A, Guo Y, Hashizume M, et al. Mortality risk attributable to high and low ambient temperature: a multicountry observational study. *The lancet* 2015; **386**(9991): 369-75.
10. Wu Y, Li S, Zhao Q, et al. Global, regional, and national burden of mortality associated with short-term temperature variability from 2000–19: a three-stage modelling study. *The Lancet Planetary Health* 2022; **6**(5): e410-e21.
11. Sera F, Armstrong B, Blangiardo M, Gasparrini A. An extended mixed-effects framework for meta-analysis. *Stat Med* 2019; **38**(29): 5429-44.
