# Supplementary material for: Impact of global short-term landscape fire sourced PM2.5 exposure on child cause-specific morbidity: a study in multiple countries and territories
Source: Nat Commun. 2025 Oct 22;16:9347. doi: 10.1038/s41467-025-64411-0 (PMC12546595; doi:10.1038/s41467-025-64411-0)
Supplement: Supplementary file 2 — Reporting summary [file 41467_2025_64411_MOESM2_ESM.pdf]

nature portfolio

Corresponding author(s): Yuming Guo and Shanshan Li

Last updated by author(s): Jul 24, 2025



# Reporting Summary

Nature Portfolio wishes to improve the reproducibility of the work that we publish. This form provides structure for consistency and transparency in reporting. For further information on Nature Portfolio policies, see our Editorial Policies and the Editorial Policy Checklist.

## Statistics

For all statistical analyses, confirm that the following items are present in the figure legend, table legend, main text, or Methods section.

| n/a | Confirmed | |
|---|---|---|
| ☒ | ☐ | The exact sample size (*n*) for each experimental group/condition, given as a discrete number and unit of measurement |
| ☐ | ☒ | A statement on whether measurements were taken from distinct samples or whether the same sample was measured repeatedly |
| ☐ | ☒ | The statistical test(s) used AND whether they are one- or two-sided<br>*Only common tests should be described solely by name; describe more complex techniques in the Methods section.* |
| ☐ | ☒ | A description of all covariates tested |
| ☐ | ☒ | A description of any assumptions or corrections, such as tests of normality and adjustment for multiple comparisons |
| ☐ | ☒ | A full description of the statistical parameters including central tendency (e.g. means) or other basic estimates (e.g. regression coefficient) AND variation (e.g. standard deviation) or associated estimates of uncertainty (e.g. confidence intervals) |
| ☐ | ☒ | For null hypothesis testing, the test statistic (e.g. *F*, *t*, *r*) with confidence intervals, effect sizes, degrees of freedom and *P* value noted<br>*Give P values as exact values whenever suitable.* |
| ☐ | ☒ | For Bayesian analysis, information on the choice of priors and Markov chain Monte Carlo settings |
| ☐ | ☒ | For hierarchical and complex designs, identification of the appropriate level for tests and full reporting of outcomes |
| ☐ | ☒ | Estimates of effect sizes (e.g. Cohen's *d*, Pearson's *r*), indicating how they were calculated |

*Our web collection on statistics for biologists contains articles on many of the points above.*

## Software and code

Policy information about availability of computer code

| | |
|---|---|
| Data collection | No software or code was used to collect the hospital admissions data. These data were obtained directly from official government sources or institutional collaborators. |
| Data analysis | All analyses were performed in R software (version 4.1.1), with dlnm, and mixmeta, splines, lubridate, and ggplot2 packages. The code used in this study is available at GitHub: https://github.com/Shuang0601/Landscape-fire-and-children-morbidity and archived on Zenodo: DOI: 10.5281/zenodo.16238692. |

For manuscripts utilizing custom algorithms or software that are central to the research but not yet described in published literature, software must be made available to editors and reviewers. We strongly encourage code deposition in a community repository (e.g. GitHub). See the Nature Portfolio guidelines for submitting code & software for further information.

## Data

Policy information about availability of data

All manuscripts must include a data availability statement. This statement should provide the following information, where applicable:

- Accession codes, unique identifiers, or web links for publicly available datasets
- A description of any restrictions on data availability
- For clinical datasets or third party data, please ensure that the statement adheres to our policy

The health and hospital admission data used in this study are derived from multiple countries. Due to ethical and legal restrictions, these data cannot be made

publicly available. The data are available under restricted access for reasons of participant confidentiality and national data-sharing agreements. Access can be requested by contacting the corresponding authors, YG (yuming.guo@monash.edu) and SL (shanshan.li@monash.edu). Requests must include a brief research proposal and documentation of relevant ethical approvals. All requests will be reviewed in a timely manner, and, if approved, data access will be granted for non-commercial research purposes for a fixed period as specified in a data-sharing agreement.

## Research involving human participants, their data, or biological material

Policy information about studies with human participants or human data. See also policy information about sex, gender (identity/presentation), and sexual orientation and race, ethnicity and racism.

| | |
|---|---|
| Reporting on sex and gender | Stratified analyses and Wald-type tests were used to evaluate potential effect modification by sex (male vs female) in the association between landscape fire-sourced PM2.5 exposure and cause-specific hospital admissions among children and adolescents. |
| Reporting on race, ethnicity, or other socially relevant groupings | Socioeconomic status was considered in stratified analyses, using both local GDP per capita and World Bank country income classifications. Wald-type tests were used to assess effect modification. |
| Population characteristics | Hospital admission data were obtained from New South Wales (Australia), Brazil, Canada, Chile, New Zealand, Taiwan, and Thailand during 2000–2019. We constructed a multi-country dataset aggregating daily hospital admissions stratified by age group (0–4, 5–9, and 10–19 years), sex (male and female), and disease cause (classified by ICD-10 codes). |
| Recruitment | This study used administrative hospital admission data collected by government agencies and health ministries in the respective countries. No direct recruitment of participants occurred. |
| Ethics oversight | This study was approved by the Monash University Human Research Ethics Committee (ID 24439). |

Note that full information on the approval of the study protocol must also be provided in the manuscript.

# Field-specific reporting

Please select the one below that is the best fit for your research. If you are not sure, read the appropriate sections before making your selection.

☐ Life sciences ☐ Behavioural & social sciences ☒ Ecological, evolutionary & environmental sciences

For a reference copy of the document with all sections, see nature.com/documents/nr-reporting-summary-flat.pdf

# Ecological, evolutionary & environmental sciences study design

All studies must disclose on these points even when the disclosure is negative.

| | |
|---|---|
| Study description | This study used daily time series data on cause-specific hospital admissions for children and adolescents from 1,012 communities in seven countries/territories (Australia, Brazil, Canada, Chile, New Zealand, Taiwan, and Thailand) from 2000 to 2019. Daily concentrations of LFS PM2.5 were estimated using a chemical transport model GEOS-Chem at a 0.25° × 0.25° resolution. Associations between LFS PM2.5 and hospital admissions were first evaluated at the community level using a distributed lag non-linear quasi-Poisson regression model, and were then pooled using a random-effect meta-analysis. Effect modification by age, sex, and socioeconomic status were also evaluated. |
| Research sample | We analysed existing datasets of daily hospital admissions among children and adolescents aged 0–19 years from 1,012 communities across seven countries/territories (Australia, Brazil, Canada, Chile, New Zealand, Taiwan, and Thailand) from 2000 to 2019. In total, 67.9 million all-cause admissions [31.4 million (46.3%) girls] were included. Each record contained information on the individual's community, age, sex, date of admission, and primary cause of hospitalisation coded according to ICD-10. These data were obtained from national health departments or official health databases. No experimental manipulation was performed. The sample represents the general paediatric and adolescent populations in the included regions. |
| Sampling strategy | This multi-country study included 1,012 communities from seven countries/territories (Australia, Brazil, Canada, Chile, New Zealand, Taiwan, and Thailand) between 2000 and 2019. Communities were selected based on the availability and completeness of daily hospital admission data for children and adolescents (aged 0–19 years), as well as reliable air pollution and meteorological data. The sampling procedure was designed to ensure wide geographic coverage and population diversity, covering both fire-prone regions (Brazil, Chile, Australia, Canada, and Thailand) and non-fire-prone regions (New Zealand and Taiwan), enabling comparative assessments of landscape fire $PM_{2.5}$ impacts across different exposure contexts. No formal sample size calculation was performed prior to the study, as this was a retrospective analysis of all available data. However, the final dataset comprised 67.9 million hospital admissions, which provides substantial statistical power to detect associations, even in subgroup and cause-specific analyses. The large sample size and broad spatial-temporal coverage enhance the robustness and generalizability of the findings. |
| Data collection | Individual hospital admission data were collected and recorded by governmental health agencies in each participating country or territory, using standardized electronic health information systems. Specifically, data were obtained from the New South Wales Admitted Patient Data Collection (APDC) in Australia (covering 100% of hospitalization records in NSW, which represents 32% of the national population), the Hospital Information System of the Unified Health System (SIH-SUS) in Brazil (covering 70–80% of hospitalizations), the Hospital Morbidity Database (HMDB) maintained by the Canadian Institute for Health Information (covering all hospitalizations), the Ministry of Health (MOH) of Chile (covering ~70% of hospitalizations), the National Minimum Dataset in New |

Zealand (covering all hospitalizations), the National Health Insurance Research Database (NHIRD) in Taiwan (covering all hospitalizations), and the Ministry of Public Health (MoPH) in Thailand (covering all hospitalizations). These data were routinely recorded by hospital staff during the admission process using electronic medical record systems, and were later compiled and quality-checked by national health authorities for research and public health surveillance purposes.For the current study, individual-level records were aggregated by age group (0–4, 5–9, and 10–19 years), sex (male or female), and disease cause according to the International Classification of Diseases, 10th Revision (ICD-10) codes. The selected cause categories included all-cause admissions, infectious diseases (A00–B99), cancer (C00–C97), diabetes (E10–E14), neurological disorders (G00–G99), cardiovascular diseases (I00–I99 and G45–G46), respiratory diseases (J00–J99), digestive diseases (K00–K93), and renal diseases (N00–N19). Data from Taiwan were not available for cardiovascular, neurological, diabetes, cancer, or renal diseases due to small case numbers. The resulting dataset comprised daily counts of cause-specific hospital admissions by age and sex in 1,012 communities across seven countries/territories between 2000 and 2019.

Timing and spatial scale

Hospital admission data were collected at a daily resolution from 2000 to 2019 across 1,012 communities in seven countries/territories. The spatial scale varied by location, including Statistical Area Level 3 (SA3) in New South Wales, Australia (83 units, 2001–2019), immediate regions in Brazil (509 units, 2007–2019), second-level administrative divisions in Canada (256 units, 2005–2019), immediate regions in Chile (15 units, 2001–2019), territories in New Zealand (66 units, 2000–2019), provinces in Thailand (77 units, 2015–2019), and municipalities in Taiwan (6 units, 2000–2018). Data were aggregated as daily counts by age, sex, and cause of hospital admission. There were no planned gaps in the data collection within each country's study period.

Data exclusions

NA

Reproducibility

All analyses were conducted using standardised code developed and validated by our research group. The analysis was performed by a single analyst following a predefined protocol. The methods used in this study have been applied in numerous peer-reviewed publications in high-impact journals, demonstrating their robustness and reliability. To ensure reproducibility, the code was executed multiple times and consistently yielded identical results. We also performed a series of sensitivity analyses to assess the robustness of our findings, including: 1) placing the knots for natural cubic spline of LFS PM2.5 at 10th and 90th or 20th and 80th centiles of the mean exposure distribution. 2) changing the df for meteorological variables: temperature from 4 to 3 and 5, RH from 3 to 4 and 5. 3) changing df for time trends from 7 to 8 and 9. All analyses produced stable effect estimates, confirming the reproducibility of the results.

Randomization

NA

Blinding

NA

Did the study involve field work? ☐ Yes ☒ No

# Reporting for specific materials, systems and methods

We require information from authors about some types of materials, experimental systems and methods used in many studies. Here, indicate whether each material, system or method listed is relevant to your study. If you are not sure if a list item applies to your research, read the appropriate section before selecting a response.

## Materials & experimental systems

| n/a | Involved in the study | |
|---|---|---|
| ☒ | ☐ | Antibodies |
| ☒ | ☐ | Eukaryotic cell lines |
| ☒ | ☐ | Palaeontology and archaeology |
| ☒ | ☐ | Animals and other organisms |
| ☒ | ☐ | Clinical data |
| ☒ | ☐ | Dual use research of concern |
| ☒ | ☐ | Plants |

## Methods

| n/a | Involved in the study | |
|---|---|---|
| ☒ | ☐ | ChIP-seq |
| ☒ | ☐ | Flow cytometry |
| ☒ | ☐ | MRI-based neuroimaging |

## Plants

Seed stocks

*Report on the source of all seed stocks or other plant material used. If applicable, state the seed stock centre and catalogue number. If plant specimens were collected from the field, describe the collection location, date and sampling procedures.*

Novel plant genotypes

*Describe the methods by which all novel plant genotypes were produced. This includes those generated by transgenic approaches, gene editing, chemical/radiation-based mutagenesis and hybridization. For transgenic lines, describe the transformation method, the number of independent lines analyzed and the generation upon which experiments were performed. For gene-edited lines, describe the editor used, the endogenous sequence targeted for editing, the targeting guide RNA sequence (if applicable) and how the editor was applied.*

Authentication

*Describe any authentication procedures for each seed stock used or novel genotype generated. Describe any experiments used to assess the effect of a mutation and, where applicable, how potential secondary effects (e.g. second site T-DNA insertions, mosiacism, off-target gene editing) were examined.*
